# Biomechanical Testing of a Calcium Phosphate-Phosphoserine–Based Mineral-Organic Adhesive for Non-invasive Fracture Repair of Mandibular Fractures in Dogs

**DOI:** 10.3389/fvets.2020.00059

**Published:** 2020-02-27

**Authors:** Alexander T. Geddes, Graham P. Thatcher, Scott Hetzel, Ronald P. McCabe, Ray Vandereby, Christopher J. Snyder

**Affiliations:** ^1^Veterinary Dentistry and Oral Surgery, Department of Surgical Sciences, School of Veterinary Medicine, University of Wisconsin-Madison, Madison, WI, United States; ^2^Department of Biostatistics and Medical Informatics, University of Wisconsin-Madison, Madison, WI, United States; ^3^Department of Orthopedics and Rehabilitation, University of Wisconsin-Madison, Madison, WI, United States

**Keywords:** non-invasive, fracture, repair, mandible, dogs, adhesive, strength, stiffness bone healing

## Abstract

Mandibular fracture repair is complicated by limited availability of bone as well as the presence of the neurovascular bundle and an abundance of tooth roots. Fractures at the location of the mandibular first molar teeth are common and it can be particularly challenging to apply stable fixation. Non-invasive fracture repair techniques utilize intraoral placement of fixation devices typically involving polymerized composites and/or interdental wiring. A novel calcium phosphate-phosphoserine–based mineral–organic adhesive was tested *ex vivo* to determine its effects on augmenting strength of different non-invasive fracture fixation techniques. This study both tested the use of mineral–organic adhesive for the purpose of stabilizing currently used non-invasive fracture repair constructs (intraoral composite splinting ± interdental wiring) and evaluated adhesive alone or with subperiosteally placed plates on buccal cortical bone surface. Aside from controls, not receiving an osteotomy along the mesial root of the mandibular first molar tooth, six treatment groups were tested to evaluate ultimate strength, stiffness, angular displacement, bending moment, and application time. All forms of fixation were found to be significantly weaker than control (*p* < 0.001). Only the control (*p* < 0.001) and mineral–organic adhesive and composite (*P* = 0.002) groups were found to be significantly stronger than wire and composite. No difference was noted in stiffness between any groups with control or wire and composite. Application times varied from the mineral–organic adhesive group (mean = 206 s) to mineral–organic adhesive and composite (mean = 1,281 s). Twenty-three fixation devices exhibited adhesive failure, 20 demonstrated cohesive failure, and 5 failed by cohesive and adhesive failure. When evaluating the ultimate strength of the fixation device groups, mineral–organic adhesive, and composite was shown to be the strongest construct. The use of resorbable bone adhesive and composite may provide a stronger fixation construct over interdental wire and composite for mandibular fracture repair in dogs.

## Introduction

Mandibular fractures are the most commonly occurring maxillofacial fractures in small animal veterinary patients (1–4). Approximately 90% of maxillofacial injuries in canines are reported to be mandibular fractures (1, 2) with 47% sustained in the area of the mandibular first molar tooth (1). In a study characterizing mandibular first molar root volumes compared with mandibular volume, relative root volume increased as patient body weight decreased (5). This predisposition for fracture helps explain the propensity for fracture occurrence at this location in small breed dogs.

Mandibular fracture repair can be particularly challenging in the caudal mandible of dogs. Muscular attachments and neurovascular structures in the caudal mandible complicate surgical exposure compared to rostral fracture repair. Anatomic structures, such as tooth roots and the inferior alveolar neurovascular bundle, severely limit locations where pilot screw holes can be created for conventional plate fixation (6). Non-invasive fracture repair techniques minimize surgical exposure of the fracture site and minimize risk of damaging or disrupting anatomic structures such as tooth roots and neurovascular structures. These non-invasive techniques have gained in popularity, due to extensive experience with clinical application of dental composites in veterinary medicine (7–15). The splints created of human dental composites and used in dogs and cats have been shown to be strong (16, 17) and clinically effective at achieving fracture union (8–14). Using the tension band principle, placement of non-invasive fracture repair constructs along the oral surface of the mandible capitalizes on the creation of a natural compressive force along the ventral surface of the mandible, thus stabilizing mandibular body fractures (15).

A previous study compared interdental wiring techniques and acrylic/composite splints determining that interdental wire with composite splint was stronger than either technique used alone (16). The increased strength of the combined techniques was noted to be particularly important when the mandibular first molar tooth crown was absent for use in fixation of experimentally induced fractures occurring at this location (17). The Stout multiple loop wiring technique provides the benefit of anchoring the wire device to multiple teeth on either side of the fracture, thus distributing the force of the fixation apparatus (18). Disadvantages of interdental wiring include inciting periodontal disease (19), prolonged anesthetic periods (17), and for application and removal and inadvertent wire sticks (20). In humans, it appears long-term consequences of these disadvantages are minimal (19–21).

To date, the availability of bone cements has been limited to non-resorbable materials that are comprised of polymethylmethacrylate, used commonly for the cementation of implants such as total hip replacements (22, 23). Infection (24) and adhesive failure (25) are inherent risks with materials not removed or resorbed. A variety of bone graft materials exist for the purpose of promoting bone formation or bone healing but lack adhesive properties and include autografts, allografts, and synthetics (alloplasts) (26). Obstacles to using techniques to enhance bone healing in veterinary patients include a lack of chemical adhesion and/or mechanical structure, limited commercial availability (bone morphogenic protein), increased surgical time (autograft collection), and cost of allografts (27). Alloplasts function to serve primarily as a scaffold for osteoblasts depositing bone (28). Depending on the chemical makeup of the particular alloplast product, some materials take more than 1 year to resorb and remain incorporated into healed bone (29). Calcium phosphate–based materials serve as a scaffold and over time are broken down into calcium and phosphate and ultimately incorporate into bone (28).

The novel calcium phosphate-phosphoserine–based mineral–organic adhesive (Tetranite® Stabilization Material; LaunchPad Medical, Lowell, MA, USA) is a mixture of the powder forms of both tetracalcium phosphate (TTCP) and phosphoserine, which is mixed in an aqueous medium. Once mixed, this material is self-setting as it cures, and it precipitates primarily as an amorphous calcium phosphate-phosphoserine phase, which creates strong bonds to the surfaces of both bone and metallic implants (30). Within days, the solid evolves into a more crystalline phase of calcium phosphate and calcium phosphoserine. The combination of properties affords a unique potential to serve as a mechanism to enable fracture fixation stabilization while also being resorbed and incorporated into healed bone. The material has been demonstrated to be safe, biocompatible, and resorbed in studies using canines (31). Applying the adhesive to the end surfaces of fractures provides an opportunity for intraoperative bony alignment and may serve as a primary or adjunctive form of fixation to facilitate bone healing.

Evaluating fracture fixation strength in the caudal aspect of the mandible is a highly appropriate, clinically relevant research question due to limited anchorage locations for non-invasive repair techniques. Fractures involving the mandibular first molar tooth may have limited interdental wiring or composite splint anchorage points caudal to this location. Experimental benchtop investigation into strengthening non-invasive repair techniques warrants exploration and analysis with the objective of effectively implementing these techniques clinically. By generating more predictable patient outcomes through the safe implementation of non-invasive repair techniques, the intent is to maximally stabilize bone without creating additional complications such as that seen with the more invasive forms of fracture repair such as the application of plates and screws. This study aims to determine whether the use of resorbable bone adhesive by itself, or in combination with other non-invasive fracture repair techniques, provides biomechanical advantage over interdental wiring and composite splinting. Results of this study may begin to elucidate the benefit of stabilizing mandibular fractures using resorbable bone adhesive as either a replacement for or an adjunct to other non-invasive fracture repair techniques.

## Materials and Methods

### Specimen Preparation

Right and left mandibles were collected from 28 medium-sized dogs (mean = 10.9 kg, range = 8.0–13.6 kg) over 1 year of age (mean = 16.7 months, range = 12–24 months). Ethical approval for this study was not required according to national legislation because the acquired specimens were humanely euthanized prior to, and for reasons unrelated to, this study. Cadaveric specimens rather than synthetic models were necessary because it replicates the clinical scenario and enamel and/or dentin is necessary for chemical adhesion and micromechanical retention of the *bis*-acryl composite to tooth structure (8, 9, 32). Only mandibles with complete dentition between the canine tooth and mandibular third molar tooth were selected. Dogs with periodontal disease greater than stage 1 (gingivitis only) were excluded, based on periodontal probing (33).

**Figure 1:**
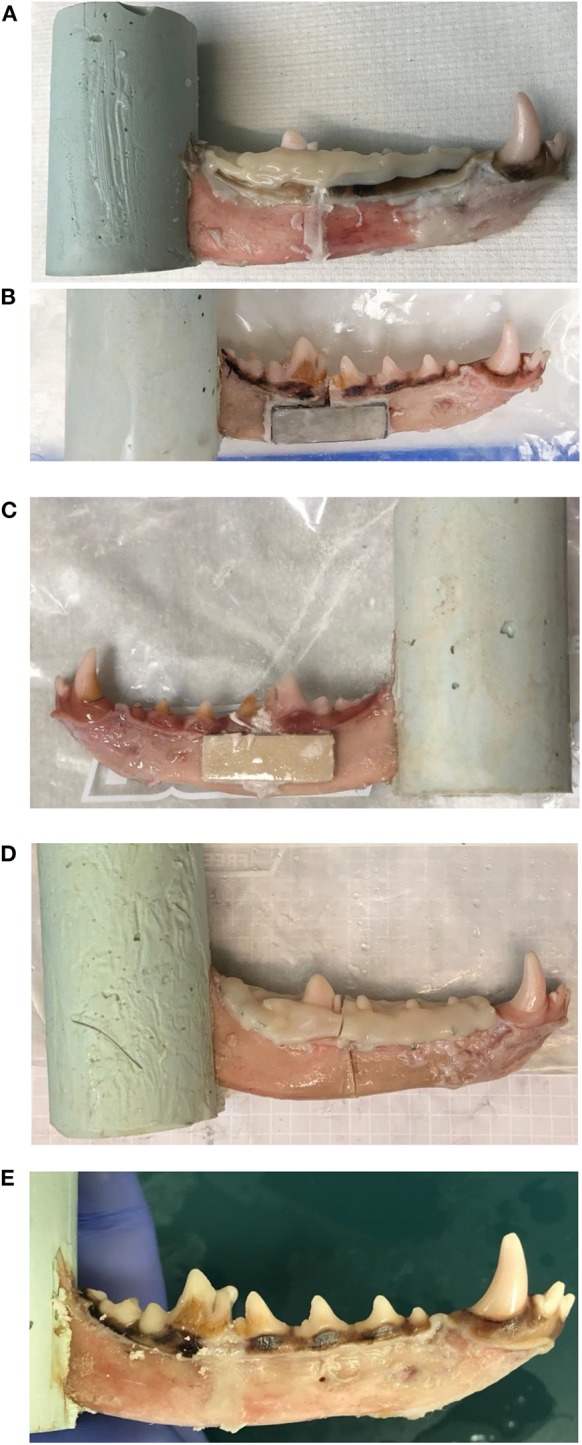
**(A–E)** Specimens demonstrating multiple repair constructs. **(A)** Adhesive and composite, **(B)** adhesive and titanium plate, **(C)** adhesive and resorbable plate, **(D)** wire and composite, and **(E)** adhesive-only are shown. **(D)** The wire and composite construct demonstrates cohesive failure of the *bis*-acryl composite splint.

Eight mandibles were randomly assigned to each of seven treatment groups: (1) mineral–organic adhesive (adhesive) applied to the ends of the cut surfaces, (2) adhesive on the cut surface and *bis*-acryl composite interdental splint, (3) interdental wire and *bis*-acryl composite splint, (4) adhesive on the cut surface and non-resorbable titanium plate adhered with adhesive, (5) adhesive on the cut surface and resorbable plate made from cured mineral–organic adhesive adhered with adhesive, (6) *bis*-acryl composite only, and (7) control mandibles (Figures 1A–E).

Fresh specimens were harvested, and all attached soft tissues were removed with the exception of attached gingiva using combination of blunt and sharp dissection. Once harvested, specimens were stored at −20°C wrapped loosely in moist paper towels to maintain hydration until preparation for testing during a single freeze–thaw cycle.

For preparation, specimens were thawed and refrigerated until potting. Specimens were potted with the ramus in polyester resin (Feather-Rite Lightweight Filler; U.S. Chemical and Plastics, Massillon, OH, USA) within a preformed mold. The mandibular body extended perpendicular from the potted ramus with the height of the mandible oriented perpendicular to the base of the mold. Osteotomies were performed in all treatment groups excluding controls. The osteotomy was created along the mesial root of the mandibular first molar tooth, perpendicular to the long axis of the mandibular body and perpendicular to the buccal bone surface using an oscillating saw (MM40 Oscillating Tool; Dremel, Racine, WI, USA) and thin kerf (0.6 mm thick) blade [MM485B, 31.7 mm Blade (0.6 mm, thick); Dremel]. Following osteotomy, specimens were returned to plastic bags submerged in 100 mL of phosphate-buffered saline (PBS) (PBS tablets; Life Technologies Corp., Carlsbad CA, USA) and placed into a 37°C circulating warming bath for 24 h prior to fixation. All fixation application times were recorded.

The interdental wiring and *bis*-acryl composite group received Stout multiple loop wiring technique [24 gauge orthopedic wire (24 g orthopedic wire; Miltex, Plainsboro NJ, USA)] applied by a single investigator (C.J.S.) extending from the first premolar tooth through the third molar tooth as previously described (15–17). A *bis*-acryl composite (Maxi Temp HP 50 mL; Henry Schein Inc., Melville, NY, USA) splint was applied as described below.

All adhesive groups received the same preparation of the material prior to placement. The mineral–organic adhesive (Tetranite® Stabilization Material; LaunchPad Medical) was provided by the manufacturer in predosed vials. The material is composed of calcium phosphate (61.5%), primarily composed of TTCP phase, and phosphoserine (38.5%), which are mixed with water and applied to the fracture site. Water for injection was added to dry powder in increments of 540 μL, followed by immediate mixing of contents for 20 s in a silicone bowl with a dental spatula. Once mixing was complete, the adhesive material was transferred into a 3 mL syringe and injected onto both fracture surfaces within 90 s. Following the reconstitution of powder with water, the adhesive was applied to the bone end surfaces and manually apposed for 210 to 270 s.

The adhesive and plate and adhesive and resorbable plate groups received additional application of respective plates to the subperiosteal bone surface. Details of the plates included use of a type 2 titanium with sand blast surface finish, with dimensions 1.0 × 3.0 × 0.163 cm, and a resorbable plate, with dimensions 1.0 × 3.0 × 0.250 cm centered over the fracture line. Following fracture reduction with adhesive, the mandible was transferred into a bag of PBS (37°C) for 300 s. At 600 s, the mandible was removed from PBS, and a second mixture of the mineral–organic adhesive was used to adhere the plate to the buccal cortical surface of the mandible. The plates were allowed to cure in an ambient environment for 120 s before being returned to PBS bag and returned to the warming bath.

The adhesive and composite group received placement of adhesive to the osteotomy end surfaces, followed by application of *bis*-acryl composite to the clinical crowns from the canine tooth to third molar tooth in the fashion described below. For the adhesive and plate, adhesive and resorbable plate, and adhesive and composite groups, a 10-min curing window at 37°C was provided between adhesive stabilization and application of secondary forms of fixation.

Treatment groups receiving *bis*-acryl composite splinting as part of two-treatment stabilization underwent placement following either interdental wiring or adhesive placement. Tooth crowns were ultrasonically scaled and polished with fine grit pumice and then etched with phosphoric acid gel (Max Etch 35% phosphoric acid etchant blue; Clinicians Choice Dental Products Inc., New Milford, CT, USA) for 20 s and rinsed with distilled water as per manufacturer's instructions. The tooth's surface was lightly air dried prior to *bis*-acryl composite application. An intraoral splint was fashioned by a single investigator (C.J.S.) using the supplied auto-mixing tips and placed on the tooth crowns extending from the first premolar tooth through third molar tooth, as previously described in a clinical fashion (16, 17). All groups involving *bis*-acryl composite were timed from the start of expressing the auto-mixed composite and were stopped when the *bis*-acryl composite was considered to be clinically cured (stable and firm to the touch).

Following adhesive application, all groups were returned into the PBS environment and maintained at 37°C for 24 ± 1 h prior to mechanical testing.

### Mechanical Testing

**Figure 2:**
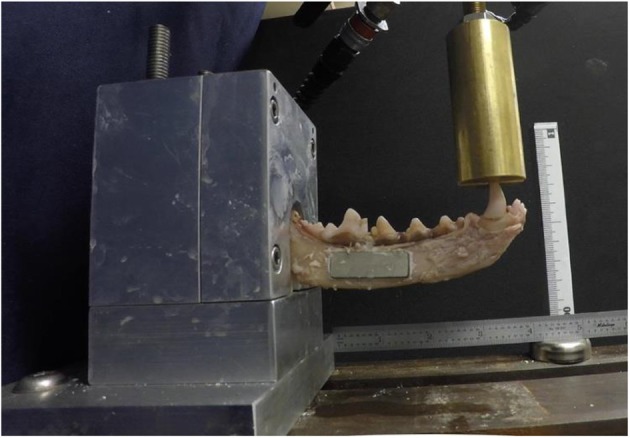
A specimen treated with endosteal resorbable bone adhesive and titanium plate is loaded in a custom jig and undergoing point force cantilevered bending with a servohydraulic testing system.

Mechanical data were measured using a servohydraulic testing system (MTS Bionix 858; MTS Systems Corp., Eden Prairie, MN, USA) in a manner previously reported (17, 34). The load application was designed to mimic cantilevered bending forces acting on the mandible. Mandibular length was measured from the canine tooth cusp to the ramus as it meets the polyester resin mold. A cantilever bending test was subsequently performed on each specimen, with a force applied to the crown of the canine tooth (Figure 2). The hydraulic actuator applied loads at a constant rate of linear deformation (10 mm/min) until fixation device failure (fracture). Actuator deformation and compressive force were recorded throughout the test. These data provided one measure of structural behavior (force vs. displacement plots). In addition, bending moments and angular displacements were calculated from these data. The moment arm was measured from the canine tooth cusp to the osteotomy location in the treatment groups and measured to where the mandibular body fractured under maximal load in the controls. Bending moment–angular displacement were calculated using the distance from the MTS grip to the canine tooth (controls) and the osteotomy to the canine tooth (treatment groups). The bending moment vs. angular displacement curves provided another measure of structural behavior. Mode of failure (adhesive or cohesive failure) was noted.

### Data Analysis

Failure was defined as the point at which either adhesive failure (gross separation of material from adhered structure) or cohesive failure (obvious fracture resulting in material breakage) occurred. This point represented the maximum force or moment. This point was identified on the force-vs.-displacement curves [load at failure (N) in Table 1] and on the bending moment–vs.–angular deformation curves [Figure 3 and bending moment at failure (Nm) in Table 3]. Linear stiffness (N/mm) was calculated from the initial linear slope of each force-vs.-displacement curve. These data are summarized in Table 2. Angular displacement at failure (degrees) was identified on each moment-vs.-angular displacement curve as the angular change of the distal mandible from uptake of load to failure. Results are summarized in Table 4.

**Table 1:** Summary of load at failure (applied to the canine tooth) with comparisons.

**Group**	**Mean (SD) {N}**	*P*-value^*^	***P*-value^^∧^^**
Wire and composite	111.6 (40.0)	Ref	<0.001^#^
Control	402.1 (82.0)	<0.001^†^	Ref
Adhesive	18.7 (4.1)	<0.001^#^	<0.001^#^
Adhesive and composite	191.6 (47.6)	0.002^†^	<0.001^#^
Adhesive and plate	40.8 (17.7)	0.009^#^	<0.001^#^
Adhesive and resorbable	19.3 (6.6)	<0.001^#^	<0.001^#^
Composite only	121.3 (40.9)	0.974	<0.001^#^

*Data are presented as mean (SD) for all treatment groups. P-values are all groups compared to wire and composite and separately all groups compared to control. P-values have a Dunnett adjustment for comparison of multiple groups to a single group*.

* *P-value indicates comparison to wire and composite*.

∧ *P-value indicates comparison to control*.

† *Group is significantly stronger to comparator*.

# *Group is significantly weaker to comparator*.

**Figure 3:**
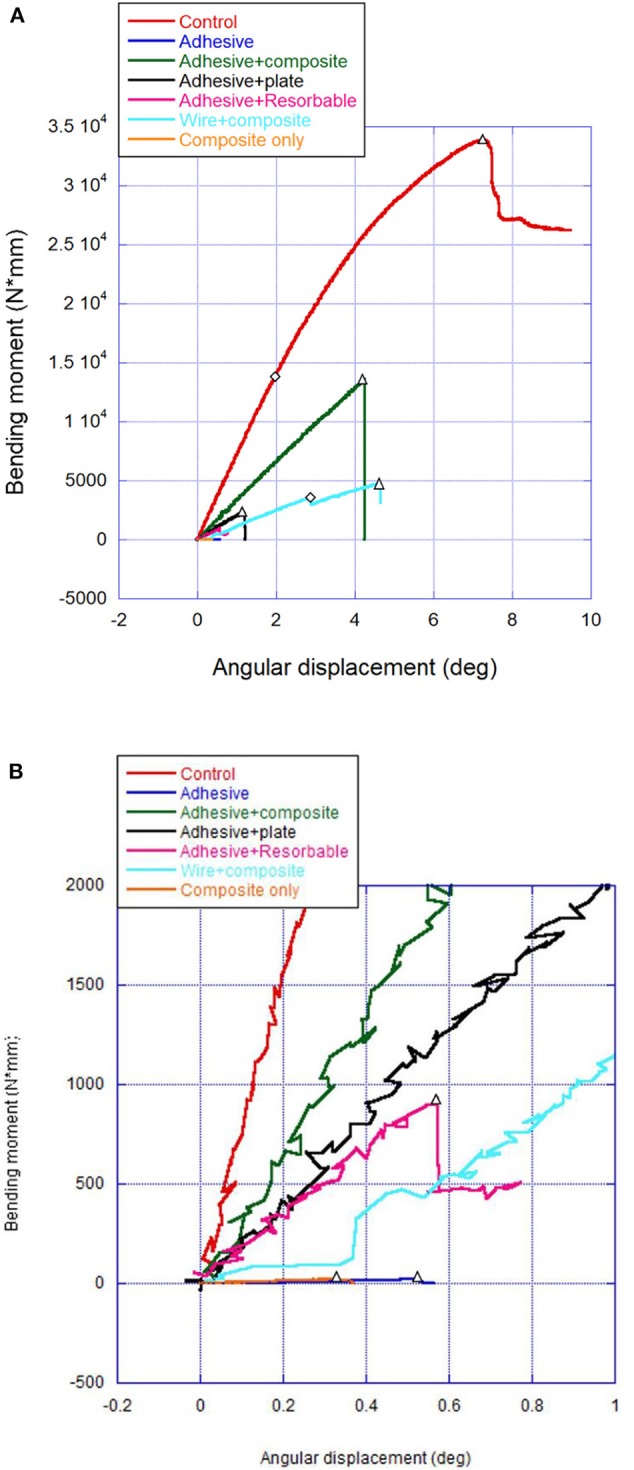
Load displacement curve. **(A,B)** Representative bending moment–angular displacement curves (**A** = full graphs, **B** = magnified insert) that depict linear limit for stiffness (diamond) when different from peak moment (triangles).

### Statistical Analysis

The average and standard deviation (SD) were calculated for load at failure, stiffness, bending moment, and angular displacement for all treatment groups.

To reduce the amount of statistical tests and hence reduce the probability of committing a type I error, we *a priori* selected to only compare the experimental groups (composite only, adhesive and resorbable, adhesive and plate, adhesive and composite, and adhesive only) to controls and then separately compare the experimental groups to wire and composite group. The control group and wire and composite group were chosen as reference groups because the control condition was an intact model of true strength potential, and wire and composite is the standard of care for repairing these types of fractures. Data were analyzed via two-sample *t*-tests with Dunnett adjustment for multiple comparisons of different groups to a single reference group. Analysis was performed to determine if a significant difference exists between groups in load to failure, stiffness, bending moment, and angular displacement. Analyses were all done using R for statistical computing, and all tests were conducted at a two-sided Dunnett adjusted 5% significance level.

## Results

Mean (±SD) load at failure for all groups and comparisons against wire and composite and controls are summarized in Table 1. All groups were significantly weaker (*P* < 0.001) than the control group (402.1 ± 82 N). The adhesive and composite group (191.6 ± 47.6 N) was the only group significantly stronger (*P* = 0.002) than the wire and composite group (111.6 ± 40.0 N). Adhesive (18.7 ± 4.1 N), adhesive and plate (40.8 ± 17.7 N), and adhesive and resorbable (19.3 ± 6.6 N) were all significantly weaker (*P* < 0.001, *P* = 0.009, *P* < 0.001, respectively) than the wire and composite group (111.6 ± 40.0 N).

Mean stiffness for all groups and comparisons against wire and composite and control groups are summarized in Table 2. No significant difference was noted between groups compared to wire and composite or control. Adhesive and composite (51.6 ± 16.0 N/mm) was noted to be stiffer at failure than wire and composite (28.3 ± 7.9 N/mm) but not considered to be significant (*P* = 0.135).

**Table 2:** Summary of stiffness (from actuator force vs. displacement) with comparisons.

**Group**	**Mean (SD) {N/mm}**	*P*-value^*^	***P*-value^∧^**
Wire and composite	28.3 (7.9)	Ref	0.567
Control	42.3 (10.4)	0.567	Ref
Adhesive	39.6 (18.0)	0.731	0.994
Adhesive and composite	51.6 (16.0)	0.135	0.832
Adhesive and plate	48.0 (22.4)	0.261	0.956
Adhesive and resorbable	30.9 (17.4)	0.996	0.72
Composite only	42.7 (38.6)	0.546	1

*Data are presented as mean (SD) for all treatment groups. P-values are all groups compared to wire and composite and separately all groups compared to control. P-values have a Dunnett adjustment for comparison of multiple groups to a single group*.

* *P-value indicates comparison to wire and composite*.

∧ *P-value indicates comparison to control*.

Mean bending moment for all groups and comparisons with wire and composite are summarized in Table 3. The bending moment for all groups was no different than wire and composite (5.6 ± 2.2 Nm) except the control group. The bending moment for the control group was 17.2 ± 13.6 Nm but was not tested because of the high loads causing the bone to bend and twist.

**Table 3:** Summary of bending moment at failure with comparisons.

**Condition**	**Mean (SD) {Nm}**	*P*-value^*^
Wire and composite	5.6 (2.2)	Ref
Control	17.2 (13.6)	<0.001^†^
Adhesive	0.9 (0.2)	0.334
Adhesive and composite	9.4 (2.6)	0.515
Adhesive and plate	2.0 (0.9)	0.580
Adhesive and resorbable	1.0 (0.3)	0.345
Composite only	5.9 (2.0)	0.999

*Data are presented as mean (SD) for all treatment groups. P-values are all groups compared to wire and composite. P-values have a Dunnett adjustment for comparison of multiple groups to a single group*.

* *P-value indicates comparison to wire and composite*.

† *Groups with a bending moment significantly greater than wire and composite*.

Mean angular displacement at failure for all groups was tested against wire and composite (Table 4). The adhesive (0.8° ± 0.7°), adhesive and plate (1.2° ± 0.4°), and adhesive and resorbable (0.9° ± 0.6°) groups all demonstrated significantly lower angular displacement (*P* ≤ 0.001) compared to the wire and composite (4.7° ± 1.6°) group. The mean angular displacement for the control group was 6.9° (±1.1°) and was dissipated over the whole length of the jaw and very different in character than the treatment groups because of the high force applied and therefore was not compared.

**Table 4:** Summary of angular displacement at failure (angular change of distal mandible from uptake of load to failure) with comparisons.

**Condition**	**Mean (SD) {**°**}**	*P*-value^*^
Wire and composite	4.7 (1.6)	Ref
Control	6.9 (1.1)	0.082
Adhesive	0.8 (0.7)	<0.001^#^
Adhesive and composite	4.9 (2.5)	0.998
Adhesive and plate	1.2 (0.4)	0.001^#^
Adhesive and resorbable	0.9 (0.6)	<0.001^#^
Composite only	5.1 (3.3)	0.97

*Data are presented as mean (SD) for all treatment groups. P-values are all groups compared to wire and composite. P-values have a Dunnett adjustment for comparison of multiple groups to a single group*.

* *P-value indicates comparison to wire and composite*.

# *Groups with an angular displacement significantly less than wire and composite*.

The application times and modes of failures are summarized in Table 5. The adhesive-only group demonstrated the shortest application time (mean 206 ± 19.6 s), whereas the adhesive and composite group showed the longest (mean 1,281 ± 49.6 s) because of the 10-min curing period between adhesive application and *bis*-acryl composite application. There was no variation in application times for adhesive and plate group and adhesive and resorbable group because of following the manufacturer's recommended time for mixing adhesive and applying adhesive. All specimens in the adhesive (8/8) and adhesive and plate (8/8) groups demonstrated adhesive failure. The wire and composite group only demonstrated cohesive (7/8) or cohesive and adhesive (1/8) failure. The composite-only group demonstrated a combination of either adhesive (4/8), cohesive (3/8), or adhesive and cohesive failure (1/8). The adhesive and resorbable plate demonstrated cohesive failure of the plate in seven of eight specimens, whereas one specimen demonstrated adhesive failure between the plate and bone. Seven of eight specimens in the adhesive and composite group demonstrated cohesive failure (four cohesive failure only, three adhesive, and cohesive failure), whereas one specimen demonstrated adhesive failure only.

**Table 5:** Summary of fixation application times and modes of failure.

		**Method of failure: adhesive**	**Method of failure: cohesive**	**Method of failure: adhesive and cohesive**
**Treatment**	**Mean application time (s) (SD)**	**(Frequency of failure within treatment group/overall)**		
Adhesive	206 (19.6)	8 (100%/16.7%)	0 (0%/0%)	0 (0%/0%)
Adhesive and composite	1281 (49.6)	1 (12.5%/2.1%)	4 (50%/8.3%)	3 (37.5%/6.3%)
Adhesive and plate	990 (0)	8 (100%/16.7%)	0 (0%/0%)	0 (0%/0%)
Adhesive and resorbable	990 (0)	0 (0%/0%)	8 (100% /16.7%)	0 (0%/0%)
Wire and composite	951 (139.1)	0 (0%/0%)	7 (87.5%/14.6%)	1 (12.5%/2.1%)
Composite only	357 (25.2)	4 (50%/8.3%)	3 (37.5%/6.3%)	1 (12.5%/2.1%)

*Mean application times for fracture fixation are reported for each group. Observational evaluation of the form of failure for each specimen is reported. Cohesive failure represents gross fragmentation of a fixation device, whereas adhesive failure represents gross delamination of the fixation device from bone or tooth. Frequency of failure occurrence is reported within the treatment group/overall*.

## Discussion

This investigation provides the first experimental data for the application of a resorbable novel calcium phosphate-phosphoserine–based mineral–organic adhesive (Tetranite® Stabilization Material; LaunchPad Medical) for the purpose of augmenting various forms of non-invasive fracture repair. Historically, the use of interdental wiring (typically Stout's multiple loop technique) in conjunction with a *bis*-acryl intraoral composite has yielded greater strength (16) and stiffness compared to composite only (17). Considering the results of wire and composite in previous forms of testing, all treatment groups in this study were evaluated against both the control group and the wire and composite treatment group.

Load at failure is a clinically relevant measurement considering the mandible's role in prehension when assessing the qualities of fixation methods. The strength of a construct is important to clinically achieve successful bone healing. Load at failure is commonly used to compare fixation methods (6, 16, 17). All testing groups in this study demonstrated a lower load to failure as compared to the control, which is consistent with the previous literature. Considering the load to failure exceeded 100 N, on average, in all three test groups utilizing *bis*-acryl composite, the composite appears to be a large contributor to the strength of these fixation devices. The use of mineral–organic adhesive as a sole form of fixation or in combination with a titanium plate or plates made of adhesive material failed at a lower load. Importantly, it was noted that a synergistic effect was achieved by combining adhesive and composite splinting. This suggests that the use of adhesive applied to the bone end surfaces in combination with composite splinting improves fixation strength when compared to interdental wiring and composite splinting.

Stiffness is an important consideration in fracture repair in order to achieve direct bone healing (35). A lack of stiffness may reduce relative stability and hence negatively impact the potential for direct bone healing. There was no difference noted between groups regarding stiffness. Despite the adhesive and composite group demonstrating the greatest stiffness, none of the constructs tested were significantly stiffer than others. The importance of stiffness may prove to be more clinically relevant in tests using cyclic loading and should be explored further.

The thickness of a material directly affects its structural stiffness (32). A single investigator (C.J.S.) applied all *bis*-acryl composite splints consistent with previous experiential clinical success. Each composite splint was fashioned in a similar manner. Stiffness is calculated from the slope of the load displacement curve (32). Materials undergoing less displacement to reach the same load demonstrate greater stiffness. For this study, the rate of displacement was fixed and directly related to time. Stiffer materials would be more appropriate for primary bone healing by stabilizing and protecting the fracture site and have been shown to ensure more advanced healing at the same time point (35). The relative flexibility of bone likely contributed to no differences between stiffnesses when comparing groups (36). Voluntary bite force in humans immediately following fracture repair with miniplating techniques demonstrates reduced forces following repair (37). Similar protective tendencies in veterinary jaw fracture patients may explain why non-invasive fracture repair techniques such as wire and composite with lower loads to failure and stiffness no different than bone may result in successful bone healing despite not demonstrating optimal biomechanical environments consistent with primary bone healing such as rigid fixation.

Bending moment describes the load placed on the construct causing angular deformation. It is a common physiological load placed upon bones, and its measurement can help determine strength of a construct (38, 39). Consistent with load to failure, the control group's bending moment was significantly greater compared to treatment groups at the moment of failure, reflecting bone's natural tendency to bend (36). Despite bending moments being significantly lower compared to controls, this may be less relevant in the clinical setting. All bending moments for treatment groups were calculated from the canine tooth cusp to fracture plane. The bending moment for controls was calculated from the canine tooth to the point where the fracture propagation extended to the alveolar crestal surface. The large load to failure for the control group likely contributed to the significant difference between bending moments of the control group compared with all treatment groups. The addition of mineral–organic adhesive to the construct contributed to the increase in bending moment, although not significant, and supports optimizing the stability of the construct. Bending moment applied to the canine tooth exerts a larger bending moment than if the load is across multiple teeth because of a larger moment arm (40). This is in contrast to *in vivo* biomechanical force application where loads are distributed across multiple teeth in a quadrant, including caudal to the fracture site. This reduces the moment arm and resultant load on the fracture site (40).

The invasiveness for application of different fracture repair techniques should be considered when selecting a fixation method. Interdental wiring is reported in the human literature to be associated with a low complication rate and minimal impact on periodontal health (19–21). Similar application techniques are used in veterinary interdental wiring. Veterinary patients create additional challenges due to the lack of bunodont dentition, which results in difficulty when placing interdental wire supragingivally. As a result, subgingival perforations are frequently necessary to successfully anchor the wiring (18). The consequences on the periodontal tissues of this technique in veterinary patients remain unreported in an objective fashion. However, clinical experience suggests that while interdental wiring is non-invasive relative to creating holes in bone that can impact tooth vitality, periodontal health is impacted. Unless interdental wire constructs can be improved to spare negative effects on periodontal health, the use of adhesives for stabilization of fractures becomes an attractive alternative, especially if it adds strength as shown in this model. The contribution of splints to inciting periodontal disease is expected, and quantification of the improvement on periodontal health through the avoidance of interdental wiring in those constructs requires investigation. Unlike the necessity to remove wire, resorbable mineral–organic adhesive has been shown to be osseointegrated and negates added time for removal.

Fracture fixation time required and ease of application of the fixation method become considerations if construct strength and stability are comparable. It is worth noting that the composite-only application time was less than half the time of the wire and composite group. The longer duration of application for the adhesive and composite group included a 10-min cure time between adhesive application to bone ends and composite placement. These 10 min were included to mimic time necessary for closure of the soft tissues between adhesive application and composite placement. Despite this technique being the slowest method to execute, the advantage of a greater load to failure over wire and composite is seemingly clinically relevant. The interdental wire and composite and adhesive and composite both require second anesthetic episodes for splint ± wire removal. The amount of time saved and resultant periodontal health without the wires have not been quantified; however, it would be expected to result in healthier periodontium and expedited splint removal in the adhesive and composite group.

Various forms of failure occurred between treatment groups. All specimens in the adhesive-only and adhesive and titanium plate groups exhibited adhesive failure. The assessment of adhesive failure in those respective groups was based on gross visualization. Adhered surfaces were assessed to have suffered adhesive failure by gross examination. Microscopic examination of the adhesive would be necessary to determine if cohesive failure existed within the cured material. All specimens in the adhesive-only group suffered adhesive failure, which is unsurprising considering the adhesive was not expected to withstand loads exceeding bone. All specimens in the adhesive and titanium plate group demonstrated adhesive failure, which is consistent with the adhesive's strong affinity to bind to metal (30). It is possible that the adhesive and titanium plate was weaker than adhesive and composite or wire and composite due to the location of fixation device, as well as a mismatched strength for adhesion between adhesive-titanium and adhesive-bone surfaces resulting in delamination of the apparatus. The tension band principle is leveraged in constructs involving use of composite material on the alveolar surface. Both the resorbable and titanium plates, placed subperiosteally, do not leverage this advantageous location and are subsequent to shear forces, which disrupted adhesion. The adhesive and resorbable plate, made of cured segments of the precast adhesive, exhibited cohesive failure in seven of eight specimens. While this was not found to be a superior form of stabilization, it does demonstrate the adhesive's ability to adhere bone to the cured material itself.

The wire and composite group demonstrated cohesive failure in seven of eight specimens, whereas one of eight specimens demonstrated adhesive failure between composite and tooth structure and cohesive failure. The composite-only group exhibited a mixture of failures including 4/8 adhesive, 3/8 cohesive, and 1/8 adhesive failure between composite and tooth structure and cohesive failure. The mean load to failure was not noted to be different (*P* = 0.974) between the wire and composite (mean = 111.6 N) and composite-only (mean = 121.3N) groups. The contribution of wire to ultimate strength appears to be insignificant in cantilevered bending tests and may have contributed to the type of failure noted in this group. Stabilization with orthopedic wire is not considered a rigid form of fixation, and additional investigation, such as the use of finite element analysis, would be necessary to determine if the distribution of forces through the wire contributes to the propensity for a large number of cohesive failures to occur. The predominately even distribution of failure types of failures in the composite-only group suggests that the adhesion between tooth and composite is stronger than the forces that the composite can withstand in tension. Despite efforts to standardize positioning of the mandible when potted, varying degrees of bending and twisting of the mandible were observed during point force loading, which may have impacted on the repair construct's ability to withstand point force loading. Bending and twisting of the mandible may be mitigated in clinical patients by contributions to stabilization of the rostral fragment across the symphysis. The adhesive and composite group had a similar number of cohesive failures compared to the composite-only group; however, the adhesive and composite group demonstrated only one adhesive failure.

When utilizing adhesives, surface area is a key consideration, and an increase in surface area would likely provide improved adhesion (41). The transverse fractures generated the smallest surface area for adhesion. This fracture confirmation may be a contributing factor to the relatively low strengths of the testing groups relying solely on the bone adhesive, or with other repair constructs that rely on the bone adhesive. Oblique fracture configurations may provide greater surface area for effective adhesion, but the shear forces acting upon unfavorable fractures may compromise this surface area advantage (42). Considering the high number of fractures through the mandibular first molar tooth (43), this testing model highlights possibly the most limited benefit of using bone adhesive for fracture fixation. The role that adhesive may play at significantly stabilizing oblique fractures, with additional surface area, could positively impact load to failure and other construct mechanical qualities.

Efforts were made to mimic the physiologic environment (hydration and temperature) that the adhesive would be applied in clinical patients. Maintaining specimen hydration in PBS and a 37°C water bath were necessary to test biomechanical properties of the mineral–organic adhesive, *bis*-acryl composite, and interdental wiring, as would be present in living patients. Time for application of fixation devices was impacted by the clinical experience of the author (C.J.S.) and may not be a representative time required to perform interdental wiring or applying composite splints for others. Furthermore, despite the fact that bite forces are reduced in human patients following mandibular fracture repair, the testing with cyclic loading may be more representative of actual functional use necessitating further studies to clearly define the biomechanical behavior of non-invasive fracture repair devices. Furthermore, the use of novel resorbable mineral–organic adhesive to further augment the strength of wire and composite fixation devices has not been defined.

Cantilevered testing mimics a force distribution to load the mandible and quantify mechanical properties of non-invasive fracture repair devices. When evaluating the mechanical properties of adhesive as a sole form of fixation or as an adjunct form of fixation, it was shown that adhesive and composite was significantly stronger than wire and composite (*P* = 0.002). None of the tested repair techniques were shown to be as strong as the control (unfractured) mandibular bone, and stiffness was noted to be no different between treatment groups. Calcium phosphate-phosphoserine–based mineral–organic adhesive tightly binds to titanium and demonstrates tight adhesion to cured TTCP–phosphoserine material. The primary form of failure for wire and composite constructs was cohesive failure of the construct, whereas mixed cohesive or adhesive failure was noted in the composite-only group. The use of mineral–organic adhesive as a method to augment the strength of composite splint fabrication for the treatment of mandibular repairs appears promising.

## Data Availability Statement

The datasets generated for this study are available on request to the corresponding author.

## Ethics Statement

Ethical approval for this study was not required according to national legislation because the acquired specimens were humanely euthanized prior to, and for reasons unrelated to, this study. Cadaveric specimens rather than synthetic models were necessary since it replicates the clinical scenario and enamel and/or dentin is necessary for chemical adhesion and micromechanical retention of the *bis*-acryl composite to tooth structure 8, 9, and 31.

## Author Contributions

CS and RV: project conception and design. SH: statistical analysis. AG, GT, and CS: initial draft of the manuscript. All authors contributed to manuscript revision, read, and approved the submitted version.

### Conflict of Interest

The authors would like to acknowledge LaunchPad Medical, LLC for donating equipment and materials in support of this project. The authors declare that the research was conducted in the absence of any commercial or financial relationships that could be construed as a potential conflict of interest.
